# The Multi-Objective Optimization Design and Hydrothermal Performance Evaluation of Anhydrous Calcium Sulfate Whisker and Polyester Fiber Compound Modified Asphalt Mixture in Hot-Humid Areas

**DOI:** 10.3390/ma16206662

**Published:** 2023-10-12

**Authors:** Taotao Fan, Qiuping Song, Chundi Si, Songkai Han

**Affiliations:** 1 School of Traffic and Transportation, Shijiazhuang Tiedao University, Shijiazhuang 050043, China; fantaotao@stdu.edu.cn (T.F.); han18533099477@163.com (S.H.); 2 State Key Laboratory of Mechanical Behavior and System Safety of Traffic Engineering Structures, Shijiazhuang Tiedao University, Shijiazhuang 050043, China; 3 Hebei Provincial Communications Planning, Design and Research Institute Co., Ltd, Shijiazhuang 050200, China; sqp900618@126.com

**Keywords:** anhydrous calcium sulfate whisker, polyester fiber, asphalt mixture, central composite circumscribed design, gray correlation grade analysis, hydrothermal performance

## Abstract

In hot and humid climates, asphalt pavements frequently encounter environmental factors such as elevated temperatures and rainfall, leading to rutting deformations and potholes, which can affect pavement performance. The primary objective of this study was to enhance the hydrothermal characteristics of asphalt mixtures through an investigation into the impact of anhydrous calcium sulfate whisker (ACSW) and polyester fiber (PF) on the hydrothermal properties of asphalt mixtures. In this paper, a central composite concatenation design (CCC) was employed to determine the optimal combination of ACSW and PF contents, as well as the asphalt aggregate ratio (AAR). Each influencing factor was assigned three levels for analysis. The evaluation indexes included dynamic stability, retained Marshall stability, and tensile strength ratio. Using the analysis methods of variance and gray correlation degree analysis, the hydrothermal properties of the asphalt mixture were examined in relation to the contents of ACSW, PF, and AAR based on the CCC results. Consequently, the optimal mix design parameters for composite modified asphalt mixture incorporating ACSW and PF were determined. The results indicated that the asphalt mixtures with hydrothermal qualities exhibited optimal performance in terms of 4.1% ARR, 11.84% ACSW, and 0.4% PF. The interaction between AAR and ACSW content had a greater effect on the dynamic stability and tensile strength ratio of the asphalt mixture, whereas the incorporation of ACSW and PF had a greater effect on the retained Marshall stability of the asphalt mixture. Among the three contributing factors, AAR exhibited the strongest relationship with the hydrothermal characteristics of the asphalt mixture, followed by the ACSW content; the correlation of PF content was the lowest. Therefore, to enhance the hydrothermal characteristics of the asphalt mixture, it is important to conduct a full evaluation of the constituents of ACSW and PF, along with the AAR in hot–humid regions.

## 1. Introduction

Asphalt pavements in different climatic regions have various performance requirements for asphalt mixtures [1,2,3,4]. In order to comprehensively analyze the performance of asphalt mixtures, it is important to have a thorough comprehension of the meteorological attributes pertaining to the specific geographical region in which the road is situated. Asphalt pavements in hot–humid areas are often influenced by high temperatures, rain, and other natural conditions and are prone to rutting, and potholes in the process of use, which result in the overall functionality and durability of the asphalt pavements being significantly compromised. Therefore, researchers have occasion to conduct research on methods that would enhance the properties of asphalt pavements to be suitable for hot–humid areas.

Current research shows that there is a certain correlation between the roadway effectiveness of modified asphalt mixtures and the proportion parameters (externally modified amount and asphalt aggregate ratio). The results presented by Hong R.B. showed that when the polyester fiber (PF) content was 0.4% and the gangue powder replacement rate was 50%, the interaction between asphalt, PF, and gangue powder caused asphalt mixtures to exhibit excellent low-temperature anti-cracking properties [5]. Zhao X.W. studied the effect of Sasobit and Deurex on the characteristics of crystalline tetra-alpha-ethylene-modifying asphalt mixes and found that 4% Sasobit +2% Deurex was the optimal amount for asphalt mixtures, and they had better mechanical properties and economic benefits [6]. P. Ahmedzade found that when the asphalt content was 5.3%, asphalt mixtures made from 8% tall oil bitumen and 6% styrene–butadiene–styrene had the best physical and mechanical properties [7]. Yang B. found that the best performance of large-particle-size-modified asphalt mixtures was achieved at elevated temperatures when the asphalt aggregate ratio was 3.23% and the rock asphalt content was 20% [8]. Zhai R.X. showed that 5% nano-CaCO_3_ and 4% SBR exhibited exceptional resistance to rutting deformation under elevated temperatures [9]. In the study conducted by Gong Y.F., it was observed that the composite-modified asphalt mixtures retained their pore properties in optimal water stability even after undergoing freeze–thaw cycles with a 3.9% basalt fiber, 5.1% nano-TiO_2_/CaCO_3_ content, and 5.67% asphalt aggregate ratio [10]. Yan K.ZH. found that 15% waste tire rubber and 4% amorphous polyolefin was the optimal content for achieving the best performance for asphalt mixtures [11]. Zhang H.T. concluded that asphalt mixtures modified using 4% SBS and 15% rubber were appropriate for asphalt pavements located in high-temperature, heavy traffic regions, and asphalt mixtures containing 4% SBS and 3% SBR were deemed suitable for cold–humid regions [12]. Cheng Y.CH. found that diatomite and basalt-fiber-composite-modified asphalt mixtures were more resistant to seasonal permafrost than the base asphalt mixtures in areas with freeze–thaw cycle damage [13]. Zhe H. demonstrated that a 2% thermoplastic polyurethane and 6% amorphous poly-alpha-olefin-modified asphalt mixtures had better rutting and moisture resistance than base asphalt mixtures [14]. Therefore, optimizing the mix design of modified asphalt mixtures to adapt to the climatic conditions in hot–humid regions has a beneficial impact on the enhancement of asphalt pavements’ resistance to high temperature and water damage.

A common method for creating a mix ratio design for externally mixed and modified asphalt is to first determine the optimum asphalt aggregate ratio of asphalt mixtures under different externally mixed mixtures using the Marshall test, and then to carry out mixture performance tests such as the wheel tracking test, low bending test, immersed Marshall test, and freeze–thaw cycle test. Asphalt mixture proportion parameters with better overall performance are then obtained according to the results of the various performance tests for asphalt mixtures under different external mixing amounts. Nevertheless, this method usually requires a large amount of experimentation, resulting in the squandering of both resources and time. Thus, researchers have conducted relevant research on convenient methods applicable to the optimization of the design parameters of asphalt mixture ratios, such as response surface design and multifactorial orthogonal design. Fan T.T. used the central composite circumscribed design (CCC) method, a type of response surface design, to optimize the design parameters of polyester fiber and anhydrous calcium sulfate whisker composite modified asphalt mixtures with better low-temperature properties [15]. Ahmed I. Nassar utilized response surface design to optimize the mixing ratio of an emulsified asphalt mixture [16]. Taher B.M. used response surface design to optimize the content of asphalt and polyethylene terephthalate in asphalt mixtures, and the results showed that the Marshall properties of the modified asphalt mixture achieved the best performance after adding 5.88% asphalt and 0.18% polyethylene terephthalate [17]. Wei Y. revealed the relationship between the bone glue/polyurethane content and the properties of an asphalt binder using response surface design, and the optimal contents were 6.848% bone glue and 2.759% polyurethane [18]. Cheng Y.CH conducted a comprehensive study on the optimization design and testing of a diatomite- and basalt-fiber-compound-modified asphalt mixture. Through the application of grey correlation grade analysis, the ideal mixing scheme was determined. The experimental findings indicated that the incorporation of 14% diatomite, 0.32% basalt fibers, and 5.45% asphalt aggregate resulted in the improved high- and low-temperature performance of the asphalt mixture [19]. Du J.CH. studied an approach for conducting grey relational regression analysis on hot-mix asphalt [20]. Lyu ZH.H. conducted an analysis of the performance of cold recycled mixtures. Additionally, an objective theory was optimized for the material content based on a multi-index-weighted grey methodology [21]. Slebi Acevedo, C.J. used multi-standard decision-making technology to study the functional and mechanical properties of polyolefin-aramid fiber and hydrated-lime-modified porous asphalt mixtures [22,23].

Therefore, this article conducts a study on the optimization of mix proportion design and hydrothermal-performance-influencing factors of the asphalt mixture with the addition of ACSW and PF. Three mix ratio design parameters (namely, ACSW content, PF content, and asphalt aggregate ratio) and three hydrothermal performance evaluation indexes (namely, dynamic stability, retained Marshall stability, and tensile strength ratio) were selected. The central composite circumscribed design (CCC) and grey correlation grade analysis (GCGA) were used to perfect the mix ratio design characteristics of modified asphalt mixtures in hot–humid areas. The effects of the design parameters on the hydrothermal performance of the asphalt mixtures were explored; meanwhile, the correlation degree between the design parameters and the hydrothermal performance indexes was evaluated using GCGA.

## 2. Materials and Methods

### 2.1. Materials

SK-90# asphalt (penetration grade 80/100) was used, and its properties are shown in Table 1. The aggregate was generated in Xianyang City, Shaanxi Province. According to Chinese standard JTG E20-2011 [24], 20 sets of asphalt mixtures were prepared with an aggregate particle size characterized by a nominal maximum of 19 mm, as shown in Table 2 and Table 3. The characteristics of ACSW and PF are listed in Table 4 and Table 5, respectively.

### 2.2. Experimental Methods

#### 2.2.1. Rutting Test

The rutting test is a commonly employed technique for assessing the durability of asphalt mixtures in terms of their ability to withstand permanent deformation when subjected to repetitive loading at elevated temperatures. According to Chinese standard T0719, the evaluation of the anti-deformation of asphalt mixtures under high temperatures was conducted using the rutting test. Specimens of 300 × 300 × 50 mm were made using the wheel-rolling method according to Chinese standard T0703. Before testing, the samples were placed in an oven at 60 °C for 5 h. Then, the rutting test was conducted under 0.7 MPa rubber wheel pressure, a 42 times/min loading rate, and a test temperature of 60 °C. The value of dynamic stability (DS) was calculated based on Equation (1).

(1) $$\mathrm{DS}=\frac{N\times (t_{1}-t_{2})}{d_{1}-d_{2}}\times c_{1}\times c_{2}$$

where *N* is the loading speed, 42 times/min; *t*_1_ and *t*_2_ are the test times, usually 45 min and 60 min, respectively; *d*_1_ and *d*_2_ are the surface rut depth of the asphalt mixture specimens in correspondence with the test times *t*_1_ and *t*_2_, mm; and *c*_1_ and *c*_2_ are the test parameters, and their values are 1.0, respectively.

#### 2.2.2. Immersed Marshall Test

The immersed Marshall test is the main method employed for ascertaining the moisture sensitivity of asphalt mixtures under high-temperature immersion. The test was carried out according to Chinese standard T0709. Eight samples were generated for each mixture using the Marshall method, and these samples were evenly divided into two groups. The first group of specimens should be soaked in water at 60 °C for 30 min and tested for the stability value *MS*_0_. The second group of specimens should be soaked in water at 60 °C for 48 h and tested for the stability value *MS*_1_. The assessment of moisture stability in the asphalt mixtures was conducted by obtaining the retained Marshall stability (RMS), as shown in Equation (2).

(2) $$\mathrm{RMS}=\frac{MS_{1}}{MS_{0}}\times 100\%$$

#### 2.2.3. Freeze–Thaw Splitting Test

The moisture stability of the titanium combination was assessed using a frozen splitting test, conducted in line with Chinese standard T0729. A total of eight samples were generated using the Marshall approach and subsequently separated into two groups, resulting in an equal distribution. A single group of samples was subjected to ambient temperatures ranging from around 20 to 25 °C. An additional set of samples underwent a freezing process for 16 h at −18 °C after water saturation. These samples were subsequently immersed in a solution at 60 °C for 24 h. Following this, the two sets of specimens were immersed in water at a temperature of 25 °C for 2 h. Subsequently, an indirect tensile test was conducted, employing an indirect tensile load with a loading rate of 50 mm/min. The tensile strength ratio (TSR) is determined by calculating the average split stretch strength before (*S*_before_) and after the freeze–thaw cycle (*S*_after_), as demonstrated in Equation (3).

(3) $$\mathrm{TSR}=\frac{S_{after}}{S_{before}}\times 100\%$$

#### 2.2.4. Grey Correlation Grade Analysis

To ascertain the most suitable composition of ACSW and PF in the asphalt mixture, the CCC results were examined using GCGA [25]. The grey correlation coefficient pertaining to the reference sequence *g_0_* = (*g_0_*(1), *g_0_*(2), *g_0_*(3)) and the comparison sequence *g_i_* = (*g_i_*(1), *g_i_*(2), *g_i_*(3)), *ξ_i_*(*m*) are defined as Equation (4).

(4) $$\xi_{i}(m)=\frac{\underset{i}{\min}\underset{m}{\min}\left|g_{0}(m)-g_{i}(m)\right|+0.5\times \underset{i}{\max}\underset{m}{\max}\left|g_{0}(m)-g_{i}(m)\right|}{\left|g_{0}(m)-g_{i}(m)\right|+0.5\times \underset{i}{\max}\underset{m}{\max}\left|g_{0}(m)-g_{i}(m)\right|}$$

where *ξ_i_*(*m*) is the correlation coefficient between the *m*-th evaluation index of the *i*-th comparison group and the associated reference group. $\underset{i}{\max}\underset{m}{\max}\left|x_{0}(m)-x_{i}(m)\right|$ is the maximum difference between the reference and comparison series. $\underset{i}{\max}\underset{m}{\max}\left|x_{0}(m)-x_{i}(m)\right|$ is the minimum difference value between the reference sequence and comparative sequences.

Then, the grey correlation grade *λ_i_* is derived by calculating the weighted sum of the grey correlation coefficients, as outlined in Equation (5).

(5) $$\lambda_{i}=Z_{D}\xi_{i}(m)+Z_{R}\xi_{i}(m)+Z_{T}\xi_{i}(m)$$

(6) $$Z_{D}+Z_{R}+Z_{T}=1$$

where *Z_D_*, *Z_R_*, and Z*_T_* represent the weighting of DS, RMS, and TSR, respectively.

### 2.3. Experimental Design

CCC, a type of response surface design, is used to assess the interaction between independent and response variables within the context of an experiment. In the CCC technique, at least two numerical inputs are required and are varied in the range of alpha (*α*) to five (−*α*, −1, 0, +1, +*α*) (*α* > 1) phases. The CCC method offers the benefit of incorporating both sequential and rotational aspects within the framework of experimental design. In this paper, CCC was chosen to evaluate the association between the independent and response variables with the aim of identifying the most suitable experimental formulation. The number of test times can be expressed as (2*^m^* + 2*m* + *n*), where *m* represents the count of independent variables and *n* is the number of center points. The value of *α* can be determined by 2*^m^*^/4^.

The experimental design employed in this study necessitates the execution of 20 experimental trials. It encompassed eight factorial points, six star points, and six center points, all of which were conducted at five levels. The design parameters were as follows: *m* = 3, *n* = 6, and *α* = 1.682. The decision to establish six center points was made in order to account for potential experimental errors by the inclusion of additional replications. Three independent variables were the asphalt aggregate ratio (AAR), ACSW content (related to asphalt-binding mass ratio), and PF content (relative to asphalt mix mass ratio), as presented in Table 6. Three response variables, DS, RMS, and TSR, were chosen to optimize the content of ACSW and PF, and the AAR of the asphalt mixture. Table 7 presents the experimental design and corresponding outcomes.

Design-Expert 10.0 software was used to perform a response model (as shown in Equation (7)) and statistical regression analysis of the experimental program to determine the best combination of independent variable levels.

(7) $$R=\mu_{0}+\sum_{i=1}^{3}\mu_{i}F_{i}+\sum_{i=1}^{2}\sum_{j=i+1}^{3}\mu_{ij}F_{i}F_{j}+\sum_{i=1}^{3}\mu_{ii}F_{i}^{2}+\eta$$

where the response variable is denoted by *R*. The coded independent parameters are represented by *F_i_* and *F_j_*. The coefficients for the constant, primary, interaction, and quadratic terms are denoted by *μ*_0_, *μ_i_*, *μ_ij_*, and *μ_ii_*, respectively. The random error is denoted by *η*.

## 3. Results and Discussion

### 3.1. Statistical Modeling

To study the interaction between different independent variables and their impact on the responsive variable, analysis of variance (ANOVA) was used. When the *p*-value was less than 0.05, there was a significant difference between the model and the factor variable. The results of the ANOVA are presented in Table 8 and Table 9, respectively.

The ANOVA results for the quadratic model of the DS are presented in the first row of Table 8. The *R*-squared value of 0.9389 and the adjusted *R*-squared value of 0.8839 indicated that the fit of the DS model was strong. Furthermore, Adeq.precision was adopted to evaluate the signal-to-noise ratio of the response model. It is worth noting that the regression model was considered optimal when the Adeq.precision value exceeded 4. Based on the findings shown in Table 8, it can be observed that the Adeq.precision of the DS model was 13.292, suggesting the favorable match of the model. The statistical significance of the DS model is supported by the *p*-value, which is less than 0.0001. The ANOVA results in Table 8 demonstrate that the models for RMS and TSR exhibited a good fit.

The significance of Lack of Fit is used to evaluate the model fit. The *p*-values of the Lack of Fit for the DS, RMS, and TSR models, as presented in Table 9, were 0.4540, 0.0552, and 0.2001, respectively, which suggests that all models were well fitted.

According to the findings presented in Table 9, the DS model revealed several statistically significant terms, which included *F*_1_, *F*_1_*F*_2_, *F*_1_*F*_3_, (*F*_1_)^2^, (*F*_2_)^2^ and (*F*_3_)^2^. The significant terms of the RMS model included *F*_2_, *F*_2_*F*_3_, (*F*_1_)^2^, and (*F*_3_)^2^, and that of the TSR model consists of the significant terms *F*_1_, *F*_3_, *F*_1_*F*_2_, (*F*_1_)^2^, (*F*_2_)^2^, and (*F*_3_)^2^. After eliminating the insignificant terms, the relation between the design parameters and response variables was represented by Equations (8)–(10).

(8) $$R_{1}=2273.89+168.64F_{1}-202.0F_{1}F_{2}+288.88F_{1}F_{3}-477.46F_{1}^{2}-143.70F_{2}^{2}-113.69F_{3}^{2}$$

(9) $$R_{2}=93.71+1.57F_{2}+0.67F_{2}F_{3}+0.59F_{1}^{2}-1.45F_{3}^{2}$$

(10) $$R_{3}=103.03+3.55F_{1}+2.10F_{3}-5.14F_{1}F_{2}-3.00F_{1}^{2}-2.24F_{2}^{2}-4.00F_{3}^{2}$$

### 3.2. Muti-Objective Optimization

#### 3.2.1. Optimization Results Based on CCC

For the hot–humid area, the target values listed in Table 10 were chosen based on Chinese standard JTG F40-2004 [26]. The larger the values of response variables, the better the anti-deformation and anti-water damage of the asphalt mixture.

**Figure 1:**
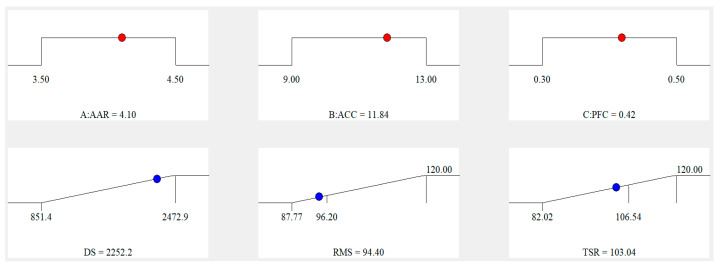
Analysis ramps of CCC.

The most suitable values of the layout parameters were determined by calculating Equations (8)–(10). The ramps of CCC analysis are depicted in Figure 1. Each ramp is associated with a specific point that represents the intended aim for both the independent and dependent variables. The meaning of the red dot in Figure 1 is the value of the ideal design parameters of asphalt mixture, and the blue dot is the value of the predicted values of hydrothermal performance of asphalt mixture. Based on the data presented in Figure 1, it can be observed that the ideal design parameters for the ACSW and PF compound-modified asphalt mixture were 4.1% AAR, 11.84% ACC, and 0.42% PFC.

Table 11 shows the optimal predicted values and laboratory values of response variables of the asphalt mixtures. The laboratory tests were conducted three times. The assessment of the deviation rate between the predicted and laboratory results was conducted using Equation (11).

(11) $$\mathrm{Deviation}\;\mathrm{rate}\;(\%)=\frac{V_{Laboratory}-V_{Predicted}}{V_{Laboratory}}\times 100\%$$

In Table 11, the deviation rates of all three response variables were lower than 2%, which showed that there was no discernible distinction between the predicted values and laboratory values. This demonstrated that it was feasible to enhance the hydrothermal characteristics of the modified asphalt mixture by optimizing its mix proportion using the CCC-RSM approach.

#### 3.2.2. Optimization Results Based on GCGA

The GCGA is a scientific methodology utilized to evaluate the correlation between components by analyzing the similarity in their developmental tendencies. This approach holds significant potential as a viable answer for research programs focused on multi-objective optimal design. The challenges in drawing accurate findings when making comparisons arise from the varying physical interpretations of the measured indicators and the lack of standardization in data dimensions.

Typically, to obtain better hydrothermal properties of the asphalt mixture, the index values of high-temperature performance and water stability are needed to reach the maximum. In GCGA, the reference series and the comparative series need to be established first. The maximum values of DS, RMS, and TSR are defined as reference series; the values of DS, RMS, and TSR are set to 2500 times/mm, 100%, and 100%, respectively. The results of 20 schemes are defined as comparative series. Secondly, the numerical dimensionless test is carried out using Equation (12), and the dimensionless results are shown in Table 12.

(12) $$x_{n}=\frac{f_{n}}{average(f_{1},f_{1},\cdots ,f_{20})}$$

where *x_n_* represents the dimensionless result of the *n*-th group, *n* = 1, 2, …, 20; and *f_n_* represents the test result of group *n*.

*ξ_i_*(*m*) of the dimensionless test results is computed based on Equation (4), as presented in Table 12. In the proportion design of the asphalt mixture in hot and humid regions, the high-temperature performance index and water stability index of the asphalt mixture are equally important. Consequently, the performance indicators are assigned equal weights in order to derive the most ideal contents of ACSW and PF, as well as the optimal asphalt aggregate ratio. The weights assigned to the performance indicators are determined as follows: *Z_D_* = 0.5, *Z_R_* = 0.25, and *Z_T_* = 0.25. The *λ* is calculated using Equation (5), as presented in Table 12. Table 13 displays the average correlation grade associated with each evaluation and level.

In the GCGA algorithm, as per its specifications, the greater the correlation rank, the closer the corresponding factor level is to the optimal value. As can be seen from Table 13, the factors correspond to the optimal ratios of ACC = 11%, PFC = 0.4%, and AAR = 4.0%. Hence, in accordance with the multi-objective results of CCC, modified asphalt mixture performs better at high temperature and has greater moisture susceptibility when the ACSW content is 11%, the PF content is 0.4%, and the asphalt aggregate ratio is 4.0%.

In addition, compared with the results of CCC and GCGA, as shown in Table 14, the deviation rates of the optimal design parameters of the modified asphalt mixtures were smaller than 7%, which revealed that it was feasible to carry out the multi-objective optimized design of asphalt mixtures using the GCGA method.

### 3.3. Hydrothermal Property Analysis

#### 3.3.1. High-Temperature Performance

**Figure 2:**
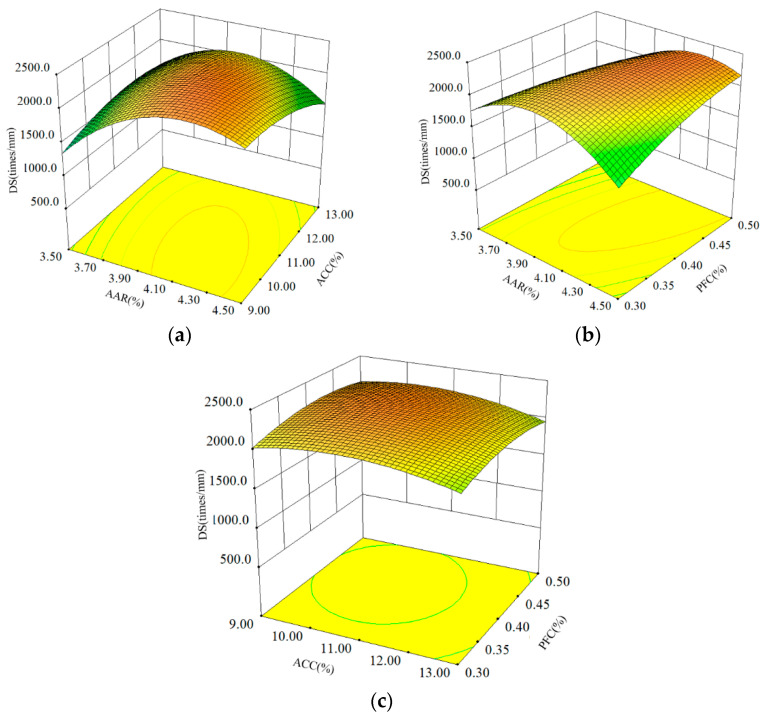
Influence of three factors on DS: (**a**) AAR and ACC; (**b**) ARR and PFC; and (**c**) ACC and PFC.

Dynamic stability (DS) was adopted to evaluate the high-temperature performance of the asphalt mixture, and the larger the DS value, the better its high-temperature performance. The DS results of the ACSW- and PF-compound-modified asphalt mixture (APCRA) at different AAR, ACC, and PFC are shown in Figure 2.

Figure 2 shows that the coupling effects of AAR and ACC, as well as AAR and PFC, exhibited more significant effects on the DS values of APCRA than the interaction between ACC and PFC, which is in accordance with the findings in Table 9. In Figure 2a,c, the DS values of APCRA first increased as the ACSW content increased from a lower level (9.0%) to a higher level (13.0%), and then stabilized with a further increase in the AAR, reaching a maximum value when the AAR was 4.1%. When the AAR was constant, the DS value of APCRA was less affected by the ACSW content. In Figure 2b, the DS value of APCRA first increased at constant PFC and then increased with AAR, and this pattern of change became more pronounced when the PFC was at a low level (0.3%) compared to a high level (0.5%).

#### 3.3.2. Moisture Susceptibility

The evaluation of moisture sensitivity in APCRA was conducted using two parameters: residual Marshall stability (RMS) and tensile strength ratio (TSR). The larger the RMS or TSR value, the better the moisture damage resistance of APCRA.

##### RMS

**Figure 3:**
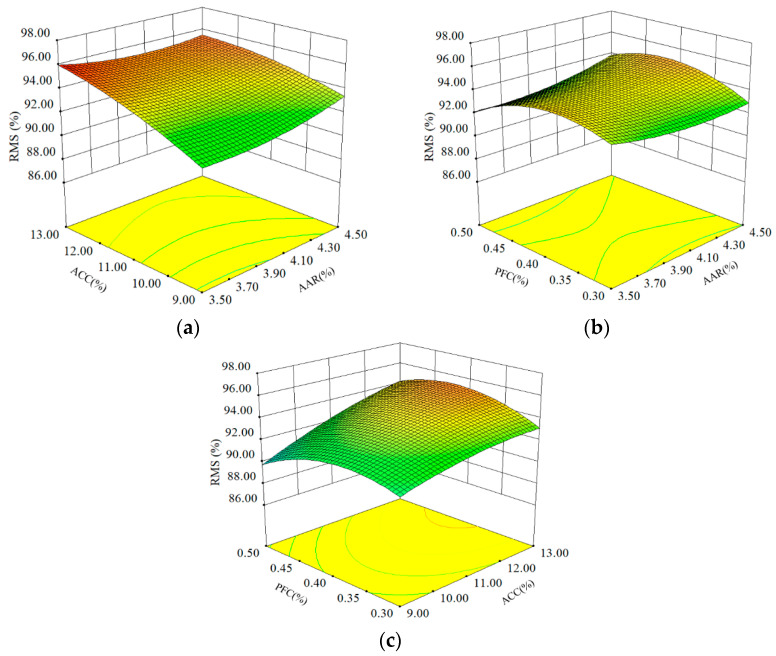
Influence of three factors on RMS: (**a**) AAR and ACC; (**b**) ARR and PFC; and (**c**) ACC and PFC.

Figure 3 shows the RMS results of APCRA at different AAR, ACC, and PFC. The coupling effect of ACC and PFC on the RMS values of APCRA was more significant than the effect of AAR and ACC or AAR and PFC on the RMS values of APC. As shown in Figure 3a,c, when the AAR or PFC was kept constant, the RMS values of APCRA increased with the increase in ACC. When the value of ACC was kept constant, the RMS values of APCRA increased and then decreased with the increase in PFC. In addition, an increase in AAR did not result in a significant change in the RMS values of APCRA.

From Figure 3a,b, the RMS values of APCRA did not change significantly with the increase in AAR at the same PFC. Meanwhile, at the same AAR, the RMS values of APCRA increased and then decreased with the increase in PFC. The RMS values of APRA reached a relative maximum when the PFC was at a medium level (0.4%) and the AAR was at a high level (4.5%).

##### TSR

**Figure 4:**
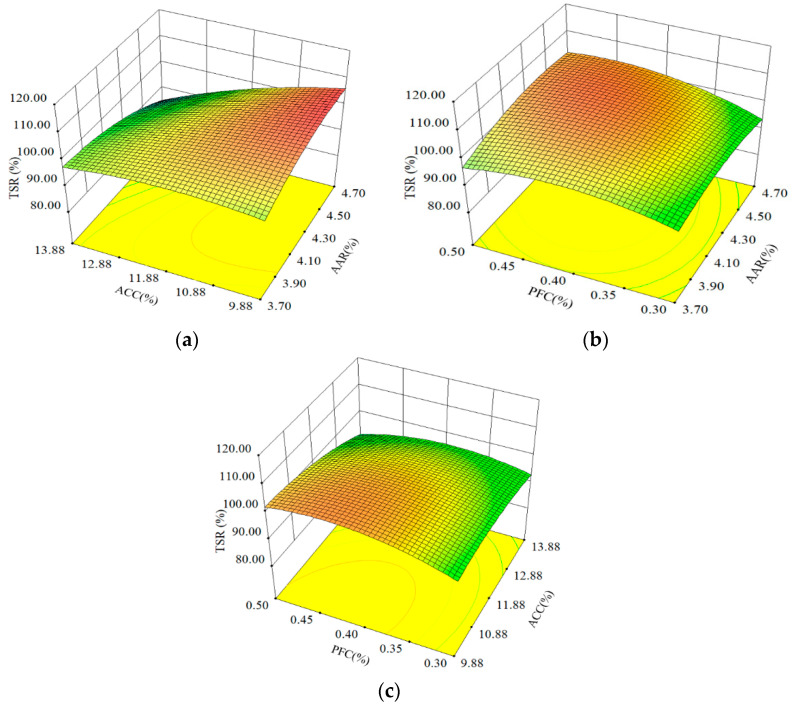
Influence of three factors on TSR: (**a**) AAR and ACC; (**b**) ARR and PFC; and (**c**) ACC and PFC.

The TSR results of APCRA at different AAR, ACC, and PFC content levels are shown in Figure 4. Figure 4a shows that the coupling relationship between ACC and AAR had a significant effect on the TSR values of APCRA. When the ACC value was at a low level (9.88%), the TSR values gradually increased with the increase in AAR, and when the ACC value was at a high level (13.88%), the TSR values gradually decreased with the increase in AAR. In addition, when the coupling values of ACC and AAR changed from 9.88% (ACC) and 3.7% (AAR) to 13.88% (ACC) and 4.7% (AAR), the TSR values increased first and then decreased.

Figure 4b shows that the TSR values of APCRA gradually increased when the coupling values of AAR and PFC changed from 0.3% (PFC) and 3.7% (AAR) to 0.5% (PFC) and 4.7% (AAR). Figure 4c illustrates that when the value of PFC was lower than 0.4%, the TSR values of APCRA increased with the increase in ACC, whereas when the value of PFC was higher than 0.4%, the TSR values of APC gradually decreased with the increase in ACC.

#### 3.3.3. Effect Degree of Factors on Performance Indexes

In this paper, GCGA was used to evaluate the correlation rank of the influencing factors with the performance indexes. The AAR, ACC, and PFC values were selected as comparison sequences, while the DS, RMS, and TSR results were adopted as reference sequences. Using Equations (4) and (12), the dimensionless results and *ξ_i_*(*m*) were calculated as shown in Table 15 and Table 16.

From Table 16, it can be observed that the grey correlation degrees of the different factors on various performance indicators were different. The grey correlation degrees of DS were as follows: AAR (0.660) > PFC (0.614) > ACC (0.593); on RMS: AAR (0.788) > ACC (0.748) > PFC (0.696); and on TSR: AAR (0.810) > ACC (0.710) > PFC (0.683). This indicates that the effect of AAR on the high-temperature and moisture sensitivity of the asphalt mixtures was more significant than that of ACC and PFC. In addition, ACC mainly affected the moisture sensitivity of the asphalt mixture, while PFC mainly affected the high-temperature performance of the asphalt mixture.

## 4. Conclusions

In this article, a multi-objective optimal proportioning design of an anhydrous calcium sulfate whisker and polyester fiber composite modified asphalt mixture was carried out using the central composite concatenation design and grey correlation grade analysis methods. The effects of anhydrous calcium sulfate whisker content, polyester fiber content, and asphalt aggregate ratio on the hydrothermal properties of the asphalt mixture were investigated using rutting tests, retained Marshall tests, and freeze–thaw splitting tests. The conclusions are as follows:

(1) The central composite concatenation design and grey correlation grade analysis methods are suitable for the quantitative study of the optimum mix ratio design of anhydrous calcium sulfate whisker and polyester fiber compound modified asphalt mixtures in hot–humid areas.
(2) The proportions of 11.8% anhydrous calcium sulfate whisker, 0.42% polyester fiber, and 4.1% asphalt aggregate can produce an asphalt mixture with better hydrothermal performance.
(3) The asphalt aggregate ratio has a greater influence on the hydrothermal performance, polyester fibers mainly affect the moisture susceptibility, and anhydrous calcium sulfate whiskers mainly affect the high-temperature properties of the asphalt mixture.

Recommendations: Feasibility research on the application of calcium sulfate whiskers as a renewable solid-waste resource in pavement materials has just begun. This paper studied the influence of calcium sulfate whiskers on the high-temperature deformation resistance and water damage resistance of an asphalt mixture and puts forward an optimization method for the mix ratio design of a modified asphalt mixture based on hygrothermal performance. However, the influence and mechanism of calcium sulfate whiskers on the low-temperature crack resistance and durability of asphalt mixtures, as well as the optimization method of the mix ratio design of modified asphalt mixtures considering physical and mechanical properties and road performance, need to be further studied.

## Figures and Tables

**Table 1:** Basic characteristics of SK-90# asphalt.

Properties	Value	Standard Value
Penetration (25 °C, 0.1 mm)	85.9	80–100
Softening point (°C)	46.5	≥45
Ductility (15 °C, cm)	>100	≥100
Viscosity (135 °C, Pa.s)	0.344	-

**Table 2:** Passing rate and physical characteristics of aggregates.

Sieve Size (mm)	19	16	13.2	9.5	4.75	2.36	1.18	0.6	0.3	0.15	0.075
Passing rate (%)	97.5	88.5	75.5	55.5	33.5	23.0	17.3	12.0	8.0	6.3	4.0
Apparent specific gravity	2.705	2.719	2.715	2.729	2.718	2.743	2.644	2.719	2.725	2.744	2.584
Bulk volume relative density	2.686	2.678	2.691	2.701	2.689	2.707	2.556	2.503	2.593	2.504	2.444
Water absorption (%)	0.263	0.555	0.335	0.380	0.400	0.489	1.314	2.746	1.866	3.502	2.222

**Table 3:** Physical characteristics of mineral powder.

Index		Results
Apparent specific gravity		2.710
Water absorption (%)		0.300
Passing rate of sieve size	<0.6 mm	100
	<0.15 mm	99.1
	<0.075 mm	90.4

**Table 4:** Properties of ACSW.

Index	Bulk Density (g/cm^3^)	Length (μm)	Diameter (μm)	Aspect Ratio	Melting Point (°C)
Value	0.1–0.4	10–200	1–4	40–100	1450

**Table 5:** Properties of PF.

Index	Diameter (μm)	Tensile Strength (MPa)	Specific Gravity (g/cm^3^)	Melting Point (°C)	Length (mm)	Elongation (%)
Value	19–21	591	1.38	259	6	10.8

**Table 6:** Experimental factors and levels of CCC.

Experimental Factors		Unit	Levels				
			−1.682	−1	0	1	1.682
*F*_1_	Asphalt aggregate ratio (AAR)	%	3.16	3.5	4	4.5	4.84
*F*_2_	ACSW content (ACC)	%	7.64	9	11	13	14.36
*F*_3_	PF content (PFC)	%	0.23	0.3	0.4	0.5	0.57

**Table 7:** Results of CCC.

No.	Experimental Variables			Response Variables		
	*F*_1_ (%)	*F*_2_ (%)	*F*_3_ (%)	DS *R*_1_ (Times/mm)	RMS *R*_2_ (%)	TSR *R*_3_ (%)
1	3.5	13	0.3	1610.1	94.25	97.02
2	4.5	9	0.5	2460.9	91.57	106.54
3	3.5	13	0.5	1123.5	93.93	93.12
4	4.0	11	0.4	2137.3	94.06	104.26
5	3.5	9	0.3	1461.7	91.59	82.02
6	4.0	11	0.4	2460.9	93.37	106.52
7	4.0	11	0.4	2113.4	93.64	101.13
8	4.0	11	0.23	1903.3	89.47	90.47
9	4.0	14.36	0.4	1862.2	95.42	93.39
10	4.0	11	0.4	2472.9	94.59	102.24
11	3.5	9	0.5	905.2	87.77	89.96
12	4.5	13	0.5	1716.6	94.77	93.35
13	4.84	11	0.4	1039.6	95.63	103.86
14	4.5	9	0.3	1707.3	92.28	96.28
15	4.0	11	0.57	2045.5	90.78	94.96
16	4.0	11	0.4	2344.8	94.35	103.33
17	3.16	11	0.4	851.4	96.20	87.23
18	4.5	13	0.3	1202.3	93.66	86.47
19	4.0	7.64	0.4	1916.8	90.65	101.96
20	4.0	11	0.4	2106.5	94.47	100.38

**Table 8:** ANOVA results of CCC.

Responses		*R*-Squared	Adj. *R*-Squared	Adeq. Precision	*F*-Value	*p*-Value	Significant
*F*_1_	*DS*	0.9389	0.8839	13.292	17.07	<0.0001	yes
*F*_2_	*RMS*	0.9252	0.8579	12.223	13.75	0.0002	yes
*F*_3_	*TSR*	0.9167	0.8418	12.643	12.23	0.0003	yes

**Table 9:** ANOVA results for experimental variables.

Responses	Factors	Sum of Squares	Degree of Freedom	Mean Square	*F*-Value	*p*-Value	Significant
DS	*F*_1_	3.884 × 10^5^	1	3.884 × 10^5^	12.08	0.0060	yes
	*F*_2_	6.953 × 10^4^	1	6.953 × 10^4^	2.16	0.1722	no
	*F*_3_	1.576 × 10^4^	1	1.576 × 10^4^	0.49	0.4998	no
	*F*_1_ *F*_2_	3.264 × 10^5^	1	3.264 × 10^5^	10.15	0.0097	yes
	*F*_1_ *F*_3_	6.676 × 10^5^	1	6.676 × 10^5^	20.77	0.0010	yes
	*F*_2_ *F*_3_	3587.04	1	3587.04	0.11	0.7453	no
	*F*_1_^2^	3.285 × 10^6^	1	3.285 × 10^6^	102.19	<0.0001	yes
	*F*_2_^2^	2.976 × 10^5^	1	2.976 × 10^5^	9.26	0.0124	yes
	*F*_3_^2^	1.863 × 10^5^	1	1.863 × 10^5^	5.79	0.0369	yes
	Lack of Fit	1.695 × 10^5^	5	3.389 × 10^4^	1.11	0.4540	no
RMS	*F*_1_	1.05	1	1.05	1.53	0.2449	no
	*F*_2_	33.60	1	33.60	48.99	<0.0001	yes
	*F*_3_	0.17	1	0.17	0.25	0.6264	no
	*F*_1_ *F*_2_	2.25	1	2.25	3.28	0.1004	no
	*F*_1_ *F*_3_	2.58	1	2.58	3.76	0.0813	no
	*F*_2_ *F*_3_	3.54	1	3.54	5.16	0.0465	yes
	*F*_1_^2^	4.31	1	4.31	6.28	0.0311	yes
	*F*_2_^2^	3.20	1	3.20	4.67	0.0560	no
	*F*_3_^2^	32.44	1	32.44	47.30	<0.0001	yes
	Lack of Fit	5.68	5	1.14	4.80	0.0552	no
TSR	*F*_1_	172.16	1	172.16	21.32	0.0010	yes
	*F*_2_	27.14	1	27.14	3.36	0.0966	no
	*F*_3_	60.44	1	60.44	7.49	0.0210	yes
	*F*_1_ *F*_2_	211.77	1	211.77	26.23	0.0004	yes
	*F*_1_ *F*_3_	21.45	1	21.45	2.66	0.1342	no
	*F*_2_ *F*_3_	28.96	1	28.96	3.59	0.0875	no
	*F*_1_^2^	129.45	1	129.45	16.03	0.0025	yes
	*F*_2_^2^	72.57	1	72.57	8.99	0.0134	yes
	*F*_3_^2^	230.32	1	230.32	28.52	0.0003	yes
	Lack of Fit	55.72	5	11.14	2.23	0.2001	no

**Table 10:** Target values of response variables.

Response	DS (*R*_1_)	RMS (*R*_2_)	TSR (*R*_3_)
Unit	times/mm	%	%
Target value	Maximize	Maximize	Maximize

**Table 11:** Validation laboratory results of CCC.

**Response**	**Unit**	**Predicted Values**	**Laboratory Values**	**Deviation Rate (%)**
AAR (*F*_1_)	%	4.10	4.10	
ACC (*F*_2_)	%	11.84	11.84	
PFC (*F*_3_)	%	0.42	0.42	
DS (*R*_1_)	times/mm	2252.2	2255.9	0.16%
RMS (*R*_2_)	%	94.40	93.53	−0.93%
TSR (*R*_3_)	%	103.04	101.25	−1.77%

**Table 12:** Analysis results of grey correlation degree.

No.	Non-Dimensional			Grey Correlation Degree			
	DS	RMS	TSR	DS	RMS	TSR	*λ*
1	0.891	1.009	0.997	0.497	0.910	0.799	0.676
2	1.362	0.980	1.094	0.986	0.862	0.958	0.948
3	0.622	1.005	0.956	0.387	0.904	0.748	0.607
4	1.183	1.007	1.071	0.717	0.907	0.915	0.814
5	0.809	0.980	0.842	0.457	0.863	0.634	0.603
6	1.362	0.999	1.094	0.986	0.894	0.958	0.956
7	1.170	1.002	1.039	0.703	0.899	0.861	0.792
8	1.053	0.957	0.929	0.599	0.828	0.717	0.686
9	1.031	1.021	0.959	0.582	0.933	0.752	0.712
10	1.369	1.012	1.050	1.000	0.917	0.879	0.949
11	0.501	0.939	0.924	0.352	0.803	0.712	0.555
12	0.950	1.014	0.959	0.530	0.920	0.751	0.683
13	0.575	1.023	1.067	0.373	0.937	0.907	0.648
14	0.945	0.987	0.989	0.527	0.875	0.789	0.680
15	1.132	0.971	0.975	0.666	0.849	0.772	0.738
16	1.298	1.010	1.031	0.869	0.912	0.898	0.887
17	0.471	1.029	0.896	0.344	0.948	0.683	0.580
18	0.665	1.002	0.888	0.401	0.899	0.675	0.594
19	1.061	0.970	1.047	0.605	0.847	0.875	0.733
20	1.166	1.011	1.031	0.699	0.914	0.849	0.790

**Table 13:** Average relational between levels of each factor and response.

ACC (%)	Relational	PFC (%)	Relational	AAR (%)	Relational
7.64	0.733	0.23	0.686	3.16	0.580
9.0	0.697	0.3	0.638	3.5	0.610
11.0	0.784	0.4	0.786	4.0	0.806
13.0	0.640	0.5	0.698	4.5	0.726
14.36	0.712	0.57	0.738	4.84	0.648

**Table 14:** Deviation rate of CCC and GCGA results.

Factors	Results of CCC (%)	Results of GCGA (%)	Deviation Rate (%)
AAR	4.1	4.0	2.44
ACC	11.8	11.0	6.78
PFC	0.42	0.4	4.76

**Table 15:** Dimensionless results.

No.	Non-Dimensional					
	AAR	ACC	PFC	DS	RMS	TSR
1	0.875	1.182	0.750	0.909	1.012	1.003
2	1.125	0.818	1.250	1.389	0.983	1.101
3	0.875	1.182	1.250	0.634	1.009	0.963
4	1.000	1.000	1.000	1.206	1.010	1.078
5	0.875	0.818	0.750	0.825	0.984	0.848
6	1.000	1.000	1.000	1.389	1.003	1.101
7	1.000	1.000	1.000	1.193	1.006	1.046
8	1.000	1.000	0.575	1.074	0.961	0.935
9	1.000	1.305	1.000	1.051	1.025	0.966
10	1.000	1.000	1.000	1.395	1.016	1.057
11	0.875	0.818	1.250	0.511	0.943	0.930
12	1.125	1.182	1.250	0.969	1.018	0.965
13	1.210	1.000	1.000	0.587	1.027	1.074
14	1.125	0.818	0.750	0.963	0.991	0.995
15	1.000	1.000	1.425	1.154	0.975	0.982
16	1.000	1.000	1.000	1.323	1.013	1.068
17	0.790	1.000	1.000	0.480	1.033	0.902
18	1.125	1.182	0.750	0.678	1.006	0.894
19	1.000	0.695	1.000	1.082	0.973	1.054
20	1.000	1.000	1.000	1.189	1.014	1.038

**Table 16:** Analysis results of grey correlation degree.

No.	DS			RMS			TSR		
	AAR	ACC	PFC	AAR	ACC	PFC	AAR	ACC	PFC
1	0.934	0.586	0.713	0.629	0.577	0.467	0.686	0.599	0.505
2	0.595	0.401	0.741	0.621	0.584	0.463	0.978	0.475	0.648
3	0.617	0.410	0.382	0.635	0.572	0.488	0.775	0.544	0.471
4	0.654	0.654	0.654	0.968	0.968	0.968	0.801	0.801	0.801
5	0.897	1.001	0.847	0.683	0.583	0.496	0.964	0.954	0.750
6	0.497	0.497	0.497	1.000	1.000	1.000	0.743	0.743	0.743
7	0.670	0.670	0.670	0.987	0.987	0.987	0.898	0.898	0.898
8	0.849	0.849	0.433	0.862	0.862	0.373	0.838	0.838	0.412
9	0.896	0.603	0.896	0.912	0.450	0.912	0.936	0.427	0.936
10	0.492	0.492	0.492	0.946	0.946	0.946	0.861	0.861	0.861
11	0.513	0.556	0.340	0.778	0.652	0.428	0.867	0.719	0.443
12	0.716	0.646	0.578	0.685	0.585	0.498	0.628	0.547	0.473
13	0.379	0.481	0.481	0.558	0.904	0.904	0.670	0.812	0.812
14	0.709	0.731	0.646	0.634	0.572	0.489	0.683	0.601	0.513
15	0.719	0.719	0.588	0.910	0.910	0.337	1.000	1.000	0.361
16	0.544	0.544	0.544	0.956	0.956	0.956	0.827	0.827	0.827
17	0.554	0.423	0.423	0.486	0.882	0.882	0.719	0.750	0.750
18	0.461	0.431	0.854	0.661	0.568	0.474	0.530	0.471	0.656
19	0.835	0.498	0.835	0.905	0.452	0.905	0.870	0.413	0.870
20	0.674	0.674	0.674	0.951	0.951	0.951	0.924	0.924	0.924
Average values	0.660	0.593	0.614	0.788	0.748	0.696	0.810	0.710	0.683

## Data Availability

Data sharing not applicable. No new data were created or analyzed in this study.
